# Human Lectins, Their Carbohydrate Affinities and Where to Find Them

**DOI:** 10.3390/biom11020188

**Published:** 2021-01-29

**Authors:** Cláudia D. Raposo, André B. Canelas, M. Teresa Barros

**Affiliations:** 1 LAQV-Requimte, Department of Chemistry, NOVA School of Science and Technology, Universidade NOVA de Lisboa, 2829-516 Caparica, Portugal; mtb@fct.unl.pt; 2 Glanbia-AgriChemWhey, Lisheen Mine, Killoran, Moyne, E41 R622 Tipperary, Ireland; canelasab@gmail.com

**Keywords:** human lectins, carbohydrate specific recognition, biological applications, targeted drug delivery systems, protein expression

## Abstract

Lectins are a class of proteins responsible for several biological roles such as cell-cell interactions, signaling pathways, and several innate immune responses against pathogens. Since lectins are able to bind to carbohydrates, they can be a viable target for targeted drug delivery systems. In fact, several lectins were approved by Food and Drug Administration for that purpose. Information about specific carbohydrate recognition by lectin receptors was gathered herein, plus the specific organs where those lectins can be found within the human body.

## 1. Introduction

**Figure 1:**
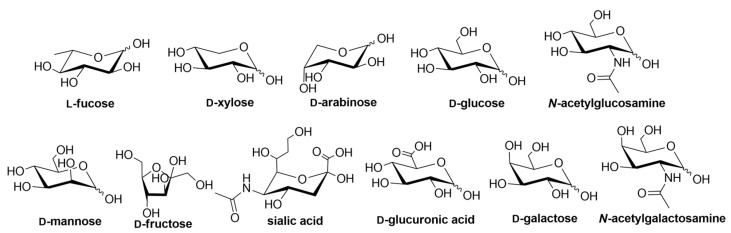
Structures of the carbohydrate building blocks found in Nature.

Lectins are an attractive class of proteins of non-immune origin that can either be free or linked to cell surfaces, and are involved in numerous biological processes, such as cell-cell interactions, signaling pathways, cell development, and immune responses [1]. Lectins selectively recognize carbohydrates and reversibly bind to them as long as the ligands are oriented in a specific manner. Some of the commonly occurring carbohydrates that are found in Nature are d-fructose, d-galactose, l-arabinose, d-xylose, d-mannose, d-glucose, d-glucosamine, d-galactosamine, l-fucose, various uronic acids, sialic acid, and their combinations to form other di- and oligosaccharides, or other biomolecules (Figure 1) [2].

Lectins in vertebrates can be classified either by their subcellular location, or by their structure. Division based on their location includes integral lectins located in membranes as structural components, or soluble lectins present in intra- and intercellular fluids, which can move freely.

Division according to lectin structure consists of several different types of lectins, such as C-type lectins (binding is Ca^2+^ dependent), I-type lectins (carbohydrate recognition domain is similar to immunoglobulins), galectin family (or S-type, which are thiol dependent), pentraxins (pentameric lectins) and P-type lectins (specific to glycoproteins containing mannose 6-phosphate) [3].

**Figure 2:**
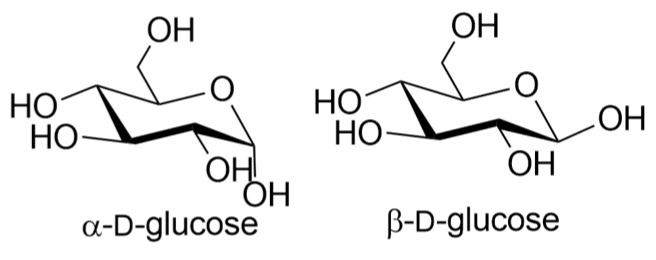
Structures of α- and β-d-glucose.

**Figure 3:**
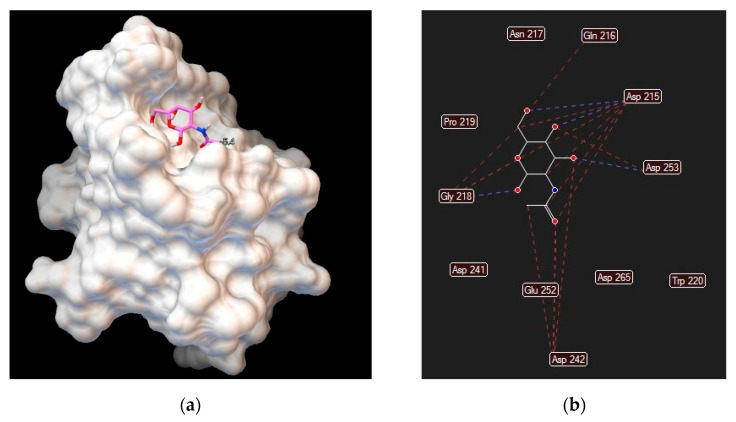
Asialoglycoprotein receptor (Protein Data Bank entry 1DV8, gene symbol ASGR1) binding interactions with *N*–acetylgalactosamine: (**a**) ligand conformation inside the binding site; (**b**) specific interactions are hydrogen bonds (blue dashed lines) and steric interactions (red dashed lines).

Different lectins have high similarity in the residues that bind to saccharides, most of which coordinate to metal ions, and water molecules. Nearly all animal lectins possess several pockets that recognize molecules other than carbohydrates, meaning that they are multivalent and can present 2 to 12 sites of interaction, allowing the binding of several ligands simultaneously. The specificity and affinity of the lectin-carbohydrate complex depends on the lectin, which can be very sensitive to the structure of the carbohydrate (e.g., mannose versus glucose, Figure 1), or to the orientation of the anomeric substituent (α versus β anomer, e.g., in Figure 2), or both. Lectin-carbohydrate interactions are achieved mainly through hydrogen bonds, van der Waals (steric interactions), and hydrophobic forces (example is given in Figure 3) [3,4].

It has been shown that the majority of lectins are conserved through evolution, suggesting that these proteins play a crucial role in the sugar-recognition activities necessary for the living process and development [5,6].

Although lectins are present in animals, plants, lichens, bacteria, and higher fungi [3], this review focuses only on human lectins for targeted drug delivery [7] purposes, their specificity towards carbohydrates and the organs where they are expressed. When referring to gene expression (or RNA expression), one means that those specific organs or cells have that specific gene coded. If active, it produces the respective protein, and one says that the protein is expressed in that organ or cell. In this review, we focus only on protein expression, since that information is the only relevant one for the development of targeted drug delivery systems. More information about carbohydrate-based nanocarriers for targeted drug delivery systems can be found elsewhere [8,9,10]. Since lectins are able to recognize and transport carbohydrates and their derivatives, lectin targeting can be relevant in the research and development of new medicines [7,11,12]. The metabolism of cancer cells, for example, is different from normal cells due to intense glycolytic activity (Warburg effect) [13]. Cancer cells require glutamine and/or glucose for cell growth, and glucose transporter isoforms 1 and 2 (gene symbols GLUT1 and GLUT2, respectively) showed an increase in activity in several tumors (gastrointestinal carcinoma, squamous cell carcinoma of the head and neck, breast carcinoma, renal cell carcinoma, gastric and ovarian cancer) [14,15].

The herein adopted lectin nomenclature is in accordance with the Human Genome Group (HUGO) Gene Nomenclature Committee. However, most common designated aliases (non-standard names) are also included (and appear first). The expression data for all lectin-coding genes was compiled from The Human Protein Atlas [16,17] and GeneCards [18] databases.

## 2. C-Type Lectins

C-type lectins are involved in the recognition of saccharides in a Ca^2+^-dependent manner but exhibit low affinities to carbohydrates, requiring multiple valencies of carbohydrate ligands to mediate signaling pathways, such as DC-SIGN2 which gene symbol is CLEC4M (Most genes carry the information to make proteins. The gene name is often used when referring to the corresponding protein). MINCLE (gene symbol CLEC4E), on the other hand, shows high affinity and can detect small numbers of glycolipids on fungal surfaces [19,20]. Most of the lectin-like domains contain some of the conserved residues required to establish the domain fold, but do not present the residues required for carbohydrate recognition [21]. The amino acid residues known to be involved in calcium-dependent sugar-binding are the EPN motif (mannose-binding), the QPD motif (for galactose binding), and the WND motif (for Ca^2+^binding) [22]. More information about glycan affinity and binding to proteins can be found elsewhere [23]. A comprehensive list of C-type lectins is presented in Table 1, divided by subfamilies that differ in the architecture of the domain [22,24], along with the carbohydrates that they recognize and the human tissues where they are expressed.

## 3. Chitolectins (or Chilectins)

There are two types of proteins that are able to recognize chitin: chitinases and chitolectins. The first ones are active proteins that bind and hydrolyze oligosaccharides, whereas the latter ones are able to bind oligosaccharides but do not hydrolyze them [76,77] and are presented in Table 2.

## 4. F-Type Lectins

F-type lectins, also called fucolectins, are characterized by an α-l-fucose recognition domain and display both unique carbohydrate- and calcium-binding sequence motifs [76]. F-type lectins are immune-recognition proteins and are presented in Table 3. Fucose is recognized by specific interactions with O5 (pyranose acetal oxygen), 3-OH and 4-OH [82], the reason why these atoms must be available to form these interactions after the synthesis of fucose derivatives.

## 5. F-Box Lectins

F-box proteins are the substrate-recognition subunits of the SCF (Skp1-Cul1-F-box protein) complex. They have an F-box domain that binds to S-phase kinase-associated protein 1 (Skp1) [84]. The F-box proteins were divided into three different classes: Fbws are those that contains WD-40 domains, Fbls containing leucine-rich repeats, and Fbxs that have either different protein-protein interaction modules or no recognizable motifs [85]. Although F-box proteins are a superfamily of proteins, only five are known to recognize N-linked glycoproteins [84] as presented in Table 4. 

## 6. Ficolins

Ficolins play an important role in innate immunity by recognizing and binding to carbohydrates present on the surface of Gram-positive and Gram-negative bacteria [89]. There are three human ficolins and they are presented in Table 5.

## 7. I-Type Lectins

I-type lectins are a subset of the immunoglobulin superfamily that specifically recognizes sialic acids and other carbohydrate ligands. Most of the members of this group of lectins are siglecs, which are type I transmembrane proteins, and can be divided into two groups: the CD33-related group that includes CD33 (siglec3) siglecs5–11, and siglec14 while the other group includes siglec1, CD22 (siglec2), MAG (siglec4) and Siglec15 [90,91]. CD33-related groups possess between 1 and 4 C-set domains and feature cytoplasmic tyrosine-based motifs involved in signaling and endocytosis. Siglec1 possesses 16 C-set domains, CD22 has 6 C-set domains and MAG presents 4 C-set domains. MAG is the only siglec not found on cells of the immune system. Members of this I-type superfamily are presented in Table 6 along with their carbohydrate ligands and protein expression. An example of a drug delivery system was developed by Spence, Greene and co-workers who developed polymeric nanoparticles of poly(lactic-co-glycolic acid) decorated with sialic acid [92,93].

## 8. L-Type Lectins

L-type lectins are distinguished from other lectins on the basis of tertiary structure, not the primary sequence, and are composed of antiparallel β-sheets connected by short loops and β-bends, usually lacking any α-helices [115]. Members of this family of lectins present different glycan-binding specificities as presented in Table 7. L-type superfamily includes Pentraxins [116,117] that require Ca^2+^ ions for ligand binding. Both LMAN1 and LMAN2 also require Ca^2+^ ions for their binding activity [115].

## 9. M-Type Lectins

M-type family of lectins consists of α-mannosidases, which are proteins involved in both the maturation and the degradation of Asn-linked oligosaccharides [127]. Members of this family, their binding affinities and protein expression are presented in Table 8.

## 10. P-Type Lectins

P-type lectins constitute a two-member family of mannose-6-phosphate receptors (Table 9) that play an essential role in the generation of functional lysosomes. The phosphate group is key to high-affinity ligand recognition by these proteins. Furthermore, optimal ligand-binding ability of M6PR is achieved in the presence of divalent cations, particularly Mn^2+^ cation [130,131].

## 11. R-Type Lectins

R-type lectins are protein-UDP acetylgalactosaminyltransferases that contain an R-type carbohydrate recognition domain, which is conserved between animal and bacterial lectins [135]. Members of this superfamily recognize Gal/GalNAc residues and are expressed in several tissues as presented in Table 10.

## 12. S-Type Lectins

S-type lectins are known nowadays as galectins and are a superfamily of proteins that show a high affinity for β-galactoside sugars (Table 11). Formerly called S-type lectins because of their sulfhydryl dependency, galectins are the most widely expressed class of lectins in all organisms. Human galectins have been classified into three major groups according to their structure: prototypical, chimeric and tandem-repeat [151,152,153].

Galectins play important roles in immune responses and promoting inflammation. They are also known for having a crucial role in cancer-causing tumor invasion, progression, metastasis and angiogenesis [154,155,156].

**Table 11:** Human S-type lectins, their carbohydrate ligands and protein epression in the organs.

Common Name (HUGO Name if Different)	Gene Symbol	Carbohydrate Preferential Affinity	Protein Expression in the Organs
**Galectin 1**			
Galectin 1	LGALS1	β-d-galactosides, poly-*N*-acetyllactosamine-enriched glycoconjugates [157,158]	Bone marrow, brain, cervix (uterine), endometrium, lymph node, ovary, parathyroid gland, placenta, smooth muscle, skin, spleen, testis, tonsil, vagina
Galectin 2	LGALS2	β-d-galactosides, lactose [159]	Appendix, colon, duodenum, gallbladder, kidney, liver, lymph node, pancreas, rectum, small intestine, spleen, tonsil
**Galectin 3**			
Galectin 3	LGALS3	β-d-galactosides, LacNAc [160]	Adipose and soft tissue, bone marrow and lymphoid tissues, brain, endocrine tissues, female tissues, gastrointestinal tract, kidney and urinary bladder, lung, male tissues, muscle tissues, pancreas, proximal digestive tract, skin
Galectin 3 binding protein	LGALS3BP	β-d-galactosides, lactose [161]	Adipose and soft tissue, bone marrow and lymphoid tissues, brain, female tissues, gastrointestinal tract, kidney and urinary bladder, lung, male tissues, muscle tissues, proximal digestive tract, skin
Galectin 4	LGALS4	β-d-galactosides, lactose [162]	Appendix, colon, duodenum, gallbladder, pancreas, rectum, small intestine, stomach
Galectin 7	LGALS7	Gal, GalNAc, Lac, LacNAc [163]	Cervix (uterine), esophagus, oral mucosa, salivary gland, skin, tonsil, vagina
Galectin 8	LGALS8	β-d-galactosides. Preferentially binds to 3′-*O*-sialylated and 3′-*O*-sulfated glycans [164]	Adipose and soft tissue, bone marrow and lymphoid tissues, brain, endocrine tissues, female tissues, gastrointestinal tract, kidney and urinary bladder, lung, male tissues, muscle tissues, pancreas, proximal digestive tract, skin
Galectin 9	LGALS9	β-d-galactosides. Forssman pentasaccharide, lactose, *N*-acetyllactosamine [165]	Adipose and soft tissue, bone marrow and lymphoid tissues, brain, endocrine tissues, female tissues, gastrointestinal tract, kidney and urinary bladder, lung, male tissues, muscle tissues, pancreas, proximal digestive tract, skin
Galectin 9B	LGALS9B	β-d-galactosides [166]	Appendix, bone marrow, breast, lymph node, spleen, tonsil
Galectin 9C	LGALS9C	β-d-galactosides [166]	Appendix, bronchus, colon, duodenum, gallbladder, lung, pancreas, spleen, stomach, tonsil
Galectin 10 (Charcot-Leyden crystal galectin, CLC)	LGALS10	Binds weakly to lactose, *N*-acetyl-d-glucosamine and d-mannose [167]	Lymph node, spleen, tonsil
Galectin 12	LGALS12	β-d-galactose and lactose [168,169]	^a)^
Galectin 13	LGALS13	*N*-acetyl-lactosamine, mannose and *N*-acetyl-galactosamine [170]. Contrary to other galectins, Galectin 13 does not bind β-d-galactosides [171]	Kidney, placenta, spleen, urinary bladder
Placental Protein 13 (Galectin 14)	LGALS14	*N*-acetyl-lactosamine [172]	Adrenal gland, colon, kidney
Galectin 16	LGALS16	*N*-acetyl-lactosamine, β-d-galactose and lactose [172]	Placenta

^a)^ Only RNA expression data available in The Human Protein Atlas [16,17] and GeneCards [18] databases.

## 13. X-Type Lectins

Intelectins (Table 12) were classified as X-type lectins because they do not have a typical lectin domain, instead, they contain a fibrinogen-like domain and a unique intelectin-specific region [173].

## 14. Orphans

Orphan lectins are those that do not belong to known lectin structural families [175]. Proteins that bind to sulfated glycosaminoglycans are usually not considered as lectins [101], however, the specific binding of these proteins to sulfated glycosaminoglycans can provide a valuable tool to develop targeted drug delivery systems. Glycosaminoglycan binding interactions with proteins were described in detail by Vallet, Clerc and Ricard-Blum [176] which information is outside of the scope of this review.

## Figures and Tables

**Table 1:** C-type superfamily, their carbohydrate ligands and protein expression in human organs.

Common Name (HUGO Name if Different)	Gene Symbol	Carbohydrate Preferential Affinity	Protein Expression in the Organs
**Proteoglycans or lecticans**			
Aggrecan	ACAN	Hyaluronic acid [25]	Cartilage, soft tissue
Brevican	BCAN	Hyaluronic acid [26,27]	Brain
Neurocan	NCAN	Hyaluronic acid [28]	Brain
Versican	VCAN	Hyaluronic acid [29]	Brain
FRAS1 related extracellular matrix 1	FREM1	^b)^	Adrenal gland, appendix, colon, duodenum, epididymis, kidney, lung, pancreas, placenta, rectum, salivary gland, small intestine, stomach, testis, tonsil, thyroid gland
**Type II transmembrane receptors**			
Blood Dendritic Cell Antigen 2 (C-type lectin domain family 4 member C)	CLEC4C	Gal-β-(1-3 or 1-4)-GlcNAc-β-(1-2)-Man trisaccharides [30,31]	Adipose and soft tissue, bone marrow and lymphoid tissues, brain, endocrine tissues, female tissues, gastrointestinal tract, kidney and urinary bladder, lung, male tissues, muscle tissues, pancreas, proximal digestive tract, skin
DC-SIGN (CD209 molecule)	CD209	High *N*-linked d-Mannose- oligosaccharides, and branched l-fucose, both with free OH-3 and OH-4. (*N*-linked glycans, *N*-acetyl-d-glucosamine, Lewis a, b, x and y) [32]	Bone marrow, lung
DC-SIGN2	CLEC4M	High *N*-linked d-Mannose- oligosaccharides, branched l-fucose, *N*-linked glycans, *N*-acetyl-d-glucosamine, Lewis a, b and y	Brain, gastrointestinal tract, lung
Dectin-2 (C-type lectin domain containing 6A)	CLEC6A	α-(1-2) or α-(1-4) mannans [33] and other high-α-d-mannose carbohydrates [34]	Blood
Dendritic cell immunoreceptor (DCIR) (C-type lectin domain family 4 member A)	CLEC4A	Mannose, fucose and weakly interacts with *N*-acetylglucosamine [35]	Bone marrow, spleen, lung
Fc fragment of IgE receptor II	FCER2	Mannose [36], immunoglobulin E, CD21, galactose [37]	Lymph node, bone marrow, spleen, appendix, tonsil, skin
Hepatic Asialoglycoprotein Receptor 1	ASGR1	Terminal β-d-galactose and *N*-acetylgalactosamine units [38]	Stomach, liver, gallbladder
Hepatic Asialoglycoprotein Receptor 2	ASGR2	Terminal β-d-galactose and *N*-acetylgalactosamine units [38]	Liver
Kupffer Cell receptor (C-type lectin domain family 4 member F)	CLEC4F	Galactose, fucose, and *N*-acetylgalactosamine [39]	Liver
Langerin (CD207 molecule)	CD207	High-mannose oligosaccharides, mannose, *N*-acetylglucosamine, fucose. Note that OH-3 and OH-4 should be free for recognition, and preferentially equatorial. *N*-acetylmannosamine showed less affinity; thereby axial derivatives should be avoided. Sulfated mannosylated glycans, keratan sulfate and β-glucans [40]	Lymph node, tonsil, skin, spleen
Liver sinusoidal epithelial cell lectin (LSECtin) (C-type lectin domain family 4 member G)	CLEC4G	Mannose, *N*-acetylglucosamine and fucose [41]	Lymph node, brain, colon, kidney, liver, testis
Macrophage Asialoglycoprotein Receptor	CLEC10A	Terminal galactose and *N*-acetylgalactosamine residues [42]	Bone marrow, brain, lymph node, oral mucosa, skin, spleen, tonsil
Macrophage C-type Lectin (MCL)	CLEC4D	Trehalose 6,6′-dimycolate, α-d-mannans18 (however it was suggested that MCL is not a carbohydrate-binding lectin) [43]	Bone marrow, lung, lymph node, spleen, tonsil
MINCLE (C-type lectin domain family 4 member E)	CLEC4E	α-mannose, trehalose-6′6-dimycolate, glucose [19]	^a)^
**Collectins**			
Collectin-K1 (collectin subfamily member 11)	COLEC11	High mannose oligosaccharides with at least a mannose-α-(1-2)-mannose residue [44]	^a)^
Collectin-L1 (collectin subfamily member 10)	COLEC10	Galactose, mannose, fucose, *N*-acetylglucosamine, *N*-acetylgalactosamine [45]	^a)^
Mannose-binding lectin 2	MBL2	Mannose, fucose, *N*-acetylglucosamine [46]	Liver
Pulmonary surfactant protein 1 (surfactant protein A1)	SFTPA1	*N*-acetylmannosamine, l-fucose, mannose, glucose, poorly to galactose. Preferentially oligosaccharides [47]	Lung
Pulmonary surfactant protein 2 (surfactant protein A2)	SFTPA2	*N*-acetylmannosamine, l-fucose, mannose, glucose, poorly to galactose. Preferentially oligosaccharides [47]	Lung
Pulmonary surfactant protein B (surfactant protein B)	SFTPB	^b)^	Lung
Pulmonary surfactant protein C (surfactant protein C)	SFTPC	Lipopolysaccharides [47]	Lung
Pulmonary surfactant protein D (surfactant protein D)	SFTPD	Maltose, glucose, mannose, poorly to galactose. Preferentially oligosaccharides [47]	Lung
Scavenger receptor with CTLD (SRCL) (collectin subfamily member 12)	COLEC12	d-galactose, l- and d-fucose, *N*-acetylgalactosamine (internalizes specifically in nurse-like cells), sialyl Lewis X, or a trisaccharide and asialo-orosomucoid (ASOR). May also play a role in the clearance of amyloid-beta in Alzheimer disease [48]	Brain, lung, placenta
**Selectins**			
Selectin E	SELE	Sialyl Lewis x, a [49]	Bone marrow, colon, nasopharynx
Selectin L	SELL	Sialyl Lewis x [50]	Appendix, bone marrow, lymph node, spleen, tonsil
Selectin P	SELP	Sialyl Lewis x [49]	Bone marrow, colon
**Natural Killer (NK)**			
C-type lectin domain family 2 member L	CLEC2L	^b)^	Brain, skeletal muscle
C-type lectin domain containing 5A	CLEC5A	Fucose, mannose, *N*-acetylglucosamine, *N*-acetylmuramic acid-β(1-4)-*N*-acetylglucosamine [51]	Blood
CD72 molecule	CD72	^b)^	Appendix, bone marrow, lymph node, spleen, tonsil
Killer cell lectin-like receptor G1	KLRG1	Mannose [52]	Appendix, cervix (uterine), colon, duodenum, small intestine, stomach, tonsil
Killer cell lectin-like receptor G2	KLRG2	^b)^	Adipose and soft tissue, bone marrow and lymphoid tissues, brain, endocrine tissues, female tissues, gastrointestinal tract, kidney and urinary bladder, lung, male tissues, muscle tissues, pancreas, proximal digestive tract, skin
CD69 molecule	CD69	Fucoidan (weak). *N*-acetylamine was reported but not supported by a second report. Does not bind glucose, galactose, mannose, fucose or *N*-acetylglucosamine [53]	Appendix, bone marrow, lymph node, spleen, tonsil
Killer cell lectin-like receptor F1	KLRF1	Predicted to not bind carbohydrates [54]	Blood
C-type lectin domain family 2 member B	CLEC2B	^b)^ Known to bind to KLRF1	Adipose and soft tissue, bone marrow and lymphoid tissues, brain, endocrine tissues, female tissues, gastrointestinal tract, kidney and urinary bladder, lung, male tissues, muscle tissues, proximal digestive tract, skin
Oxidized low-density lipoprotein receptor 1	OLR1	Predicted to not bind to carbohydrates [55]	^a)^
Killer cell lectin-like receptor D1	KLRD1	α-(2-3)-linked NeuAc on multi-antennary *N*-glycan, heparin, sulfate-containing polysaccharides [56]	^a)^
C-type lectin domain family 1 member A	CLEC1A	^b)^ [57]	^a)^
C-type lectin domain family 1 member B	CLEC1B	Predicted to not bind to carbohydrates [58]	^a)^
C-type lectin domain family 12 member B	CLEC12B	^b)^	^a)^
C-type lectin-like 1	CLECL1	Predicted to not bind to carbohydrates [21]	^a)^
C-type lectin domain family 12 member A	CLEC12A	^b)^	Bone marrow, lung, spleen
DNGR (C-type lectin domain containing 9A)	CLEC9A	Specific interactions were not discovered yet, although it is known that this lectin binds to α-actin filaments and β-spectrin [59]	^a)^
C-type lectin domain family 2 member A	CLEC2A	^b)^	Skin
Dectin-1 (C-type lectin domain containing 7A)	CLEC7A	β-(1-3)- and β-(1-6)-d-Glycans (neither mono- or short oligosaccharides/polymers are recognized) [60]	Blood, bone marrow
C-type lectin domain family 2 member D	CLEC2D	High molecular weight sulfated glycosaminoglycans [61]	Adipose and soft tissue, bone marrow and lymphoid tissues, brain, endocrine tissues, female tissues, gastrointestinal tract, kidney and urinary bladder, lung, male tissues, muscle tissues, pancreas, proximal digestive tract, skin
Killer cell lectin-like receptor B1	KLRB1	Terminal Gal-α-(1-3)-Gal, N-acetyllactosamine. [62] Sucrose octasulphate [63]	Adipose and soft tissue, bone marrow and lymphoid tissues, brain, endocrine tissues, female tissues, gastrointestinal tract, kidney and urinary bladder, lung, male tissues, muscle tissues, pancreas, proximal digestive tract, skin
Killer cell lectin-like receptor C1	KLRC1	^b)^	^a)^
Killer cell lectin-like receptor C2	KLRC2	^b)^	^a)^
Killer cell lectin-like receptor C3	KLRC3	^b)^	Colon, duodenum, small intestine, stomach, tonsil
Killer cell lectin-like receptor C4	KLRC4	^b)^	^a)^
Killer cell lectin-like receptor K1	KLRK1	α-(2-3)-NeuAc-containing *N*-glycans [64], heparin, heparan sulfate [56]	Appendix, lymph node, spleen, tonsil
**Macrophage Mannose Receptor (MMR)**			
Endo180 (Mannose receptor C type 2)	MRC2	Mannose, fucose, *N*-acetylglucosamine [65]	Adipose and soft tissue, bone marrow and lymphoid tissues, brain, endocrine tissues, female tissues, gastrointestinal tract, kidney and urinary bladder, lung, male tissues, muscle tissues, pancreas, proximal digestive tract, skin
Lymphocyte antigen 75	LY75	Predicted to not bind carbohydrates [65]	Appendix, breast, bronchus, cervix (uterine), duodenum, endometrium, fallopian tube, gallbladder, liver, lung, lymph node, nasopharynx, pancreas, placenta, rectum, spleen, stomach, thyroid gland, tonsil, urinary bladder,
Mannose receptor C-type 1 ^c)^	MRC1	Mannose, fucose, glucose, *N*-acetylglucosamine [66] (C-type) 4-*O*-sulphated GalNAc (R-type)	Colon, endometrium, kidney, lung, rectum, skin, soft tissue, testis
Phospholipase A2 receptor	PLA2R1	Predicted to not bind carbohydrates [65] but known to bind collagen	Kidney
**Free C-type Lectin Domains (CTLDs)**			
C-type lectin domain containing 19A	CLEC19A	^b)^	^a)^
Lithostathine-alpha (Regenerating family member 1 alpha)	REG1A	^b)^	Duodenum, pancreas, small intestine, stomach
Lithostathine-beta (Regenerating family member 1 beta)	REG1B	^b)^	Duodenum, pancreas, small intestine, stomach
Regenerating family member 3 alpha	REG3A	Peptidoglycan (binding affinity increases with the length of the carbohydrate moiety) [67]	Appendix, duodenum, skin, small intestine, stomach
Regenerating family member 3 gamma	REG3G	Peptidoglycan [67]	^a)^
Regenerating family member 4	REG4	Mannans, heparin [67]	Appendix, colon, duodenum, rectum, small intestine
**Type I receptors**			
Chondrolectin	CHODL	^b)^ [68]	Appendix, colon, duodenum, rectum, small intestine, testis
Layilin	LAYN	Hyaluronan [69]	Adipose and soft tissue, bone marrow and lymphoid tissues, brain, endocrine tissues, female tissues, gastrointestinal tract, kidney and urinary bladder, lung, male tissues, muscle tissues, pancreas, proximal digestive tract, skin
**Tetranectin**			
Cartilage-derived C-type lectin (C-type lectin domain family 3 member A)	CLEC3A	Expected to bind sulfated polysaccharides such as heparin [70]	^a)^
Stem cell growth factor (SCGF) (C-type lectin domain containing 11A)	CLEC11A	^b)^	Bone marrow, soft tissue
Tetranectin (C-type lectin domain family 3 member B)	CLEC3B	Sulfated polysaccharides such as heparin [70]	^a)^
**Polycystin**			
Polycystin 1 like 3, transient receptor potential channel interacting	PKD1L3	Predicted to not bind carbohydrates	^a)^
Polycystin 1, transient receptor potential channel interacting	PKD1	Predicted to bind galactosyl and glucosyl residues. Might bind oligosaccharides with mannosyl moieties [71]	Adipose and soft tissue, bone marrow and lymphoid tissues, brain, endocrine tissues, female tissues, gastrointestinal tract, kidney and urinary bladder, lung, male tissues, pancreas, proximal digestive tract, skin
**Attractin**			
Attractin	ATRN	^b)^	Adipose and soft tissue, bone marrow and lymphoid tissues, brain, endocrine tissues, female tissues, gastrointestinal tract, kidney and urinary bladder, lung, male tissues, pancreas, proximal digestive tract, skin
Attractin-like 1	ATRNL1	^b)^	^a)^
**CTLD/acidic neck**			
CD302 molecule	CD302	^b)^ [72]	^a)^
Proteoglycan 2, pro eosinophil major basic protein	PRG2	Heparin [73]	Bone marrow, placenta
Proteoglycan 3, pro eosinophil major basic protein 2	PRG3	^b)^	Bone marrow
**Endosialin**			
CD93 molecule	CD93	^b)^	Bone marrow, brain, colon, kidney, lung, spleen
C-type lectin domain containing 14A	CLEC14A	^b)^	Appendix, brain, cervix (uterine), colon, duodenum, esophagus, gallbladder, heart muscle, kidney, lung, pancreas, prostate, rectum, skin, small intestine, stomach, testis
Endosialin (CD248 molecule)	CD248	^b)^	Adipose and soft tissue, bone marrow and lymphoid tissues, brain, female tissues, gastrointestinal tract, kidney and urinary bladder, muscle tissues, pancreas, skin
Thrombomodulin	THBD	^b)^	Cervix (uterine), colon, esophagus, lymph node, oral mucosa, placenta, skin, tonsil, urinary bladder, vagina
**Others**			
C-type lectin domain family 18 member A	CLEC18A	Fucoidan, β-glucans, β-galactans [74]	^a)^
Prolectin (C-type lectin domain containing 17A)	CLEC17A	Terminal α-d-mannose and fucose residues [75]	Appendix, lymph node, spleen, stomach, tonsil
DiGeorge syndrome critical region gene 2	DGCR2	^b)^	Pancreas
FRAS1 related extracellular matrix 1	FREM1	^b)^	Adrenal gland, appendix, colon, duodenum, epididymis, kidney, lung, pancreas, placenta, rectum, salivary gland, small intestine, stomach, testis, tonsil, thyroid gland

^a)^ Only RNA expression data available in The Human Protein Atlas [16,17] and GeneCards [18] databases. ^b)^ Carbohydrate moieties recognized by this protein have not been discovered yet. ^c)^ FDA-approved drug target.

**Table 2:** Human chitolectins (also called chilectins), their carbohydrate ligands and protein expression in the organs.

Common Name (HUGO Name if Different)	Gene Symbol	Carbohydrate Preferential Affinity	Protein Expression in the Organs
Chitinase 3 like 1	CHI3L1	Chitin [78]	^a)^
Chitinase 3 like 2	CHI3L2	Chitooligosaccharides ((GlcNAc)_5_ and (GlcNAc)_6_ showed the highest affinities) [79]	Adipose and soft tissue, bone marrow and lymphoid tissues, brain, endocrine tissues, female tissues, gastrointestinal tract, kidney and urinary bladder, lung, male tissues, proximal digestive tract
Oviductin (Oviductal glycoprotein 1)	OVGP1	Chitin [80]	Fallopian tube
Stabilin-1 interacting chitinase-like protein	SI-CLP	GalNAc, GlcNAc, ribose, mannose. Prefers to bind oligosaccharides with a four-sugar ring core [81]	^a)^

^a)^ Only RNA expression data available in The Human Protein Atlas [16,17] and GeneCards [18] databases.

**Table 3:** Human f-type lectins, their carbohydrate ligands and protein expression in the organs.

Common Name (HUGO Name if Different)	Gene Symbol	Carbohydrate Preferential Affinity	Protein Expression in the Organs
Coagulation factor V ^a)^	F5	Fucose [83]	^b)^
APC, WNT signalling pathway regulator	APC	^c)^	Adipose and soft tissue, bone marrow and lymphoid tissues, brain, endocrine tissues, female tissues, gastrointestinal tract, kidney and urinary bladder, lung, male tissues, muscle tissues, pancreas, proximal digestive tract, skin

^a)^ FDA-approved drug target. ^b)^ Only RNA expression data available in The Human Protein Atlas [16,17] and GeneCards [18] databases. ^c)^ Carbohydrate moieties recognized by this protein have not been discovered yet.

**Table 4:** Human F-box lectins, their carbohydrate ligands and protein expression in the organs.

Common Name (HUGO Name if Different)	Gene Symbol	Carbohydrate Preferential Affinity	Protein Expression in the Organs
Cyclin F	CCNF	^a)^	Appendix, bone marrow, lung, lymph node, skin, spleen, tonsil
F-box protein 2	FBXO2	*N*-acetylglucosamine disaccharide chitobiose [86]	Breast, ovary, pancreas
F-box protein 3	FBXO3	^a)^	^b)^
F-box protein 4	FBXO4	^a)^	^b)^
F-box protein 5	FBXO5	^a)^	Adipose and soft tissue, bone marrow and lymphoid tissues, brain, endocrine tissues, female tissues, gastrointestinal tract, kidney and urinary bladder, lung, male tissues, muscle tissues, pancreas, proximal digestive tract, skin
F-box protein 6	FBXO6	High-mannose glycoproteins [87]	Adipose and soft tissue, bone marrow and lymphoid tissues, brain, endocrine tissues, female tissues, gastrointestinal tract, kidney and urinary bladder, lung, male tissues, muscle tissues, pancreas, proximal digestive tract, skin
F-box protein 7	FBXO7	^a)^	Adipose and soft tissue, bone marrow and lymphoid tissues, brain, endocrine tissues, female tissues, gastrointestinal tract, kidney and urinary bladder, lung, male tissues, muscle tissues, pancreas, proximal digestive tract, skin
F-box protein 8	FBXO8	^a)^	Bone marrow and lymphoid tissues, brain, endocrine tissues, female tissues, gastrointestinal tract, kidney and urinary bladder, lung, male tissues, pancreas, proximal digestive tract, skin
F-box protein 9	FBXO9	^a)^	^b)^
F-box protein 10	FBXO10	^a)^	Cervix (uterine), colon, duodenum, endometrium, fallopian tube, lung, prostate, rectum, seminal vesicle, small intestine, testis
F-box protein 11	FBXO11	^a)^	^b)^
F-box protein 15	FBXO15	^a)^	^b)^
F-box protein 16	FBXO16	^a)^	Adipose and soft tissue, bone marrow and lymphoid tissues, brain, endocrine tissues, female tissues, gastrointestinal tract, kidney and urinary bladder, lung, male tissues, muscle tissues, pancreas, proximal digestive tract, skin
F-box protein 17	FBXO17	Sulfated and galactose-terminated glycoproteins [88]	^b)^
F-box protein, helicase, 18	FBXO18	^a)^	Adipose and soft tissue, bone marrow and lymphoid tissues, brain, endocrine tissues, female tissues, gastrointestinal tract, kidney and urinary bladder, lung, male tissues, muscle tissues, pancreas, proximal digestive tract, skin
LIM domain 7	LMO7	^a)^	^b)^
F-box protein 21	FBXO21	^a)^	Adipose and soft tissue, bone marrow and lymphoid tissues, brain, endocrine tissues, female tissues, gastrointestinal tract, kidney and urinary bladder, lung, male tissues, muscle tissues, proximal digestive tract, skin
F-box protein 22	FBXO22	^a)^	^b)^
Tetraspanin 17	TSPAN17	^a)^	^b)^
F-box protein 24	FBXO24	^a)^	^b)^
F-box protein 25	FBXO25	^a)^	^b)^
F-box protein 27	FBXO27	^a)^	Adipose and soft tissue, bone marrow and lymphoid tissues, brain, endocrine tissues, female tissues, gastrointestinal tract, kidney and urinary bladder, lung, male tissues, muscle tissues, proximal digestive tract, skin
F-box protein 28	FBXO28	^a)^	^b)^
F-box protein 30	FBXO30	^a)^	^b)^
F-box protein 31	FBXO31	^a)^	Adipose and soft tissue, bone marrow and lymphoid tissues, brain, endocrine tissues, female tissues, gastrointestinal tract, kidney and urinary bladder, lung, male tissues, muscle tissues, proximal digestive tract, skin
F-box protein 32	FBXO32	^a)^	^b)^
F-box protein 33	FBXO33	^a)^	Adipose and soft tissue, bone marrow and lymphoid tissues, brain, endocrine tissues, female tissues, gastrointestinal tract, kidney and urinary bladder, lung, male tissues, muscle tissues, pancreas, proximal digestive tract, skin
F-box protein 34	FBXO34	^a)^	Adrenal gland, bronchus, colon, epididymis, endometrium, gallbladder, placenta, seminal vesicle, skeletal muscle, skin, stomach, testis, thyroid gland
F-box protein 36	FBXO36	^a)^	^b)^
F-box protein 38	FBXO38	^a)^	^b)^
F-box protein 39	FBXO39	^a)^	^b)^
F-box protein 40	FBXO40	^a)^	^b)^
F-box protein 41	FBXO41	^a)^	^b)^
F-box protein 42	FBXO42	^a)^	Bone marrow and lymphoid tissues, brain, endocrine tissues, female tissues, gastrointestinal tract, kidney and urinary bladder, lung, male tissues, pancreas
F-box protein 43	FBXO43	^a)^	^b)^
F-box protein 44	FBXO44	^a)^	Adipose and soft tissue, bone marrow and lymphoid tissues, brain, endocrine tissues, female tissues, gastrointestinal tract, kidney and urinary bladder, lung, male tissues, muscle tissues, pancreas, proximal digestive tract, skin
F-box protein 45	FBXO45	^a)^	^b)^
F-box protein 46	FBXO46	^a)^	^b)^
F-box protein 47	FBXO47	^a)^	^b)^
F-box protein 48	FBXO48	^a)^	Esophagus, kidney, oral mucosa, parathyroid gland, skin, stomach

^a)^ Carbohydrate moieties recognized by this protein have not been discovered yet. ^b)^ Only RNA expression data available in The Human Protein Atlas [16,17] and GeneCards [18] databases.

**Table 5:** Human ficolins, their carbohydrate ligands and protein expression in the organs.

Common Name (HUGO Name if Different)	Gene Symbol	Carbohydrate Preferential Affinity	Protein Expression in the Organs
Ficolin 1	FCN1	GlcNAc, GalNAc; sialic acid [89]	^a)^
Ficolin 2	FCN2	GlcNAc (acetyl group); β-(1-3)-d-glucan [89]	^a)^
Ficolin 3	FCN3	*N*-acetylglucose; *N*-acetylgalactose, fucose, lipopolysaccharides [89]	^a)^

^a)^ Only RNA expression data available in The Human Protein Atlas [16,17] and GeneCards [18] databases.

**Table 6:** Human I-type lectins, their carbohydrate ligands and protein expression in the organs.

Common Name (HUGO Name if Different)	Gene Symbol	Carbohydrate Preferential Affinity	Protein Expression in the Organs
Siglecl1 (Sialic acid binding Ig like lectin 1)	SIGLEC1	α-(2-3)-Sialic acid, α-(2-6)-Sialic acid, α-(2-8)-Sialic acid [94]	Bone marrow, lung
Siglec2 (CD22 molecule) ^a)^	CD22	α-(2-6)-Sialic acid [95,96]	Appendix, lymph node, spleen, tonsil
Siglec3 (CD33 molecule)	CD33	α-(2-6)-Sialic acid, α-(2-3)-Sialic acid [97]	Appendix, bone marrow, lung, lymph node, skin, spleen, tonsil
Siglec4a, MAG (Myelin associated glycoprotein)	MAG	α-(2-3)-Sialic acid [98]	Brain
Siglec5 (Sialic acid binding Ig like lectin 5)	SIGLEC5	α-(2-3)-Sialic acid, α-(2-6)-Sialic acid, α-(2-8)-Sialic acid [99]	Bone marrow, lymph node, placenta, spleen, tonsil
Siglec6 (Sialic acid binding Ig like lectin 6)	SIGLEC6	Sialic acid-α-(2-6)-*N*-acetylgalactosamine (Sialyl-Tn) [100]	Placenta
Siglec7	SIGLEC7	α-(2-6)-Sialic acid, α-(2-8)-Sialic acid, α-(2-3)-Sialic acid [101] and disialogangliosides [102,103,104]	^b)^
Siglec8	SIGLEC8	α-(2-3)-Sialic acid, α-(2-6)-Sialic acid [105]	Adipose and soft tissue, bone marrow and lymphoid tissues, brain, endocrine tissues, female tissues, gastrointestinal tract, kidney and urinary bladder, lung, male tissues, muscle tissues, pancreas, proximal digestive tract, skin
Siglec9 (Sialic acid binding Ig like lectin 9)	SIGLEC9	α-(2-3)-Sialic acid, Sialyl Lewis x, α-(2-6)-Sialic acid, α-(2-8)-Sialic acid [106]	Adipose and soft tissue, bone marrow and lymphoid tissues, brain, endocrine tissues, female tissues, gastrointestinal tract, kidney and urinary bladder, lung, male tissues, muscle tissues, pancreas, proximal digestive tract, skin
Siglec10 (Sialic acid binding Ig like lectin 10)	SIGLEC10	α-(2-3)-Sialic acid, α-(2-6)-Sialic acid [107]	Appendix, bone marrow, lymph node, soft tissue, spleen, tonsil
Siglec11 (Sialic acid binding Ig like lectin 11)	SIGLEC11	α-(2-8)-Sialic acid [101]	^b)^
Siglec14 (Sialic acid binding Ig like lectin 14)	SIGLEC14	Sialic acid- α-(2-6)-*N*-acetylgalactosamine (Sialyl-Tn), *N*-acetylneuraminic acid [108]	Adipose and soft tissue, bone marrow and lymphoid tissues, brain, endocrine tissues, gastrointestinal tract, kidney and urinary bladder, lung, male tissues, muscle tissues, pancreas, proximal digestive tract, skin
Siglec15 (Sialic acid binding Ig like lectin 15)	SIGLEC15	Sialyl-Tn [109]	^b)^
CD2 molecule ^a)^	CD2	*N*-glycans with fucose [110]	Appendix, lymph node, spleen, tonsil
CD83 molecule	CD83	Sialic acid [111]	Appendix, bone marrow, lung, lymph node, spleen, tonsil
Intercellular adhesion molecule 1	ICAM1	Hyaluronan [112]	Appendix, bone marrow, brain, endometrium, fallopian tube, kidney, lung, lymph node, spleen, testis, tonsil
L1 cell adhesion molecule	L1CAM	α-(2-3)-Sialic acid [113]	Adipose and soft tissue, bone marrow and lymphoid tissues, brain, female tissues, gastrointestinal tract, kidney and urinary bladder, lung, male tissues, muscle tissues, proximal digestive tract, skin
Myelin protein zero	MPZ	SO_4_^–^ –3GlucA-β-(1-3)-Gal-β-(1–4)-GlcNAc (HNK-1 antigen) [101]	Bronchus, esophagus, fallopian tube, small intestine, soft tissue, stomach, testis
Neural cell adhesion molecule 1	NCAM1	High *N*-linked d-mannose [114]	Brain, colon, hearth muscle, pancreas, smooth muscle, soft tissue, thyroid gland
Neural cell adhesion molecule 2	NCAM2	^c)^	Brain, bronchus, colon, duodenum, gallbladder, ovary, rectum, small intestine, soft tissue, testis

^a)^ FDA-approved drug target. ^b)^ Only RNA expression data available in The Human Protein Atlas [16,17] and GeneCards [18] databases. ^c)^ Carbohydrate moieties recognized by this protein have not been discovered yet.

**Table 7:** Human L-type lectins, their carbohydrate ligands and protein expression in the organs.

Common Name (HUGO Name if Different)	Gene Symbol	Carbohydrate Preferential Affinity	Protein Expression in the Organs
Calnexin	CANX	Non-reducing glucose residues in an oligosaccharide (Glc(Man)_9_(GlcNAc)_2_) [118]	Adipose and soft tissue, bone marrow and lymphoid tissues, brain, endocrine tissues, female tissues, gastrointestinal tract, kidney and urinary bladder, lung, male tissues, muscle tissues, pancreas, proximal digestive tract, skin
Calreticulin	CALR	Non-reducing glucose residues in an oligosaccharide (Glc(Man)_9_(GlcNAc)_2_) [119]	Bone marrow and lymphoid tissues, brain, endocrine tissues, female tissues, gastrointestinal tract, kidney and urinary bladder, lung, male tissues, pancreas, skin
Calreticulin 3	CALR3	^a)^	Testis
Lectin, mannose-binding 1	LMAN1	α-(1-2) mannans with free OH-3, OH-4 and OH-6 [120]	Adipose and soft tissue, bone marrow and lymphoid tissues, brain, endocrine tissues, female tissues, gastrointestinal tract, kidney and urinary bladder, lung, male tissues, muscle tissues, pancreas, proximal digestive tract, skin
Lectin, mannose-binding 1 like	LMAN1L	^a)^	^b)^
Lectin, mannose-binding 2	LMAN2	High α-(1-2) mannans, Low affinity for d-glucose and *N*-acetylglucosamine [121]	Bone marrow and lymphoid tissues, brain, endocrine tissues, female tissues, gastrointestinal tract, kidney and urinary bladder, lung, male tissues, pancreas
Lectin, mannose-binding 2 like	LMAN2L	α-(1-2) trimannose [122]	Adipose and soft tissue, bone marrow and lymphoid tissues, brain, endocrine tissues, female tissues, gastrointestinal tract, kidney and urinary bladder, lung, male tissues, muscle tissues, pancreas, proximal digestive tract, skin
Adhesion G protein-coupled receptor D1	ADGRD1	^a)^	Adipose and soft tissue, bone marrow and lymphoid tissues, brain, endocrine tissues, female tissues, gastrointestinal tract, kidney and urinary bladder, lung, male tissues, muscle tissues, pancreas, proximal digestive tract, skin
Adhesion G protein-coupled receptor D2	ADGRD2	^a)^	^b)^
Amyloid P component, serum	APCS	Heparin, dextran sulfate proteoglycans [123]	^b)^
C-reactive protein	CRP	Galactose 6-phosphate, Gal-β-(1-3)-GalNAc, Gal-β-(1-4)-GalNAc, Gal-β-(1-4)-Gal-β-(1-4)-GlcNAc, other phosphate-containing ligands [124,125]	Liver, gallbladder, soft tissue
Neuronal pentraxin 1	NPTX1	^a)^	Brain, testis
Neuronal pentraxin 2	NPTX2	^a)^	Adrenal gland, brain, pancreas, pituitary gland, testis
Neuronal pentraxin receptor	NPTXR	^a)^	Brain
Pentraxin 3	PTX3	Heparin [126]	^b)^
Sushi, von Willebrand factor type A, EGF and pentraxin domain containing 1	SVEP1	^a)^	Adipose and soft tissue, bone marrow and lymphoid tissues, brain, endocrine tissues, female tissues, gastrointestinal tract, kidney and urinary bladder, lung, male tissues, muscle tissues, pancreas

^a)^ Carbohydrate moieties recognized by this protein have not been discovered yet. ^b)^ Only RNA expression data available in The Human Protein Atlas [16,17] and GeneCards [18] databases.

**Table 8:** Human M-type lectins, their carbohydrate ligands and protein expression in the organs.

Common Name (HUGO Name if Different)	Gene Symbol	Carbohydrate Preferential Affinity	Protein Expression in the Organs
Mannosidase alpha class 1A member 1	MAN1A1	α-(1-2)-mannans [128,129]	Adipose and soft tissue, bone marrow and lymphoid tissues, brain, endocrine tissues, female tissues, gastrointestinal tract, kidney and urinary bladder, lung, male tissues, muscle tissues, pancreas, proximal digestive tract, skin
Mannosidase alpha class 1A member 2	MAN1A2	α-(1-2)-mannans [128,129]	Bone marrow and lymphoid tissues, brain, endocrine tissues, female tissues, gastrointestinal tract, kidney and urinary bladder, lung, male tissues, muscle tissues, pancreas, proximal digestive tract, skin
Mannosidase alpha class 1B member 1	MAN1B1	α-(1-2)-mannans [128,129]	Adipose and soft tissue, bone marrow and lymphoid tissues, brain, endocrine tissues, female tissues, gastrointestinal tract, kidney and urinary bladder, lung, male tissues, muscle tissues, pancreas, proximal digestive tract, skin
Mannosidase alpha class 1C member 1	MAN1C1	α-(1-2)-mannans [128,129]	Bone marrow and lymphoid tissues, brain, endocrine tissues, female tissues, gastrointestinal tract, kidney and urinary bladder, lung, male tissues, muscle tissues, pancreas

**Table 9:** Human P-type lectins, their carbohydrate ligands and protein expression in the organs.

Common Name (HUGO Name if Different)	Gene Symbol	Carbohydrate Preferential Affinity	Protein Expression in the Organs
Mannose-6-phosphate receptor, cation dependent ^a)^	M6PR	Mannose-6-phosphate residues [132,133]	Adipose and soft tissue, bone marrow and lymphoid tissues, brain, endocrine tissues, female tissues, gastrointestinal tract, kidney and urinary bladder, lung, male tissues, muscle tissues, pancreas, proximal digestive tract, skin
Insulin-like growth factor 2 receptor	IGF2R	Mannose-6-phosphate residues (either α or β). Mannose-6-phosphate analogues with carboxylate or malonate groups [134]	^b)^

^a)^ FDA-approved drug target. ^b)^ Only RNA expression data available in The Human Protein Atlas [16,17] and GeneCards [18] databases.

**Table 10:** Human R-type lectins, their carbohydrate ligands and protein expression in the organs.

Common Name (HUGO Name if Different)	Gene Symbol	Carbohydrate Preferential Affinity	Protein Expression in the Organs
Polypeptide *N*-acetylgalactosaminyltransferase 1	GALNT1	GalNAc [136]	Adipose and soft tissue, bone marrow and lymphoid tissues, brain, endocrine tissues, female tissues, gastrointestinal tract, kidney and urinary bladder, lung, male tissues, muscle tissues, pancreas, proximal digestive tract, skin
Polypeptide *N*-acetylgalactosaminyltransferase 2	GALNT2	GalNAc [136,137]	Bone marrow and lymphoid tissues, brain, endocrine tissues, female tissues, gastrointestinal tract, kidney and urinary bladder, lung, male tissues, muscle tissues, pancreas, proximal digestive tract, skin
Polypeptide *N*-acetylgalactosaminyltransferase 3	GALNT3	GalNAc [136]	Adipose and soft tissue, bone marrow and lymphoid tissues, brain, endocrine tissues, female tissues, gastrointestinal tract, kidney and urinary bladder, lung, male tissues, muscle tissues, pancreas, proximal digestive tract, skin
Polypeptide *N*-acetylgalactosaminyltransferase 4	GALNT4	GalNAc, GalNAc-glycosylated substrates [136,138]	^a)^
Polypeptide *N*-acetylgalactosaminyltransferase 5	GALNT5	GalNAc [136]	Appendix, bronchus, cervix (uterine), colon, duodenum, esophagus, gallbladder, lung, oral mucosa, rectum, salivary gland, small intestine, stomach, tonsil, vagina
Polypeptide *N*-acetylgalactosaminyltransferase 6	GALNT6	GalNAc [136]	Bone marrow and lymphoid tissues, brain, endocrine tissues, female tissues, gastrointestinal tract, kidney and urinary bladder, lung, male tissues, muscle tissues, pancreas, proximal digestive tract, skin
Polypeptide *N*-acetylgalactosaminyltransferase 7	GALNT7	GalNAc, GalNAc-glycosylated substrates [100]	Bone marrow and lymphoid tissues, brain, endocrine tissues, female tissues, gastrointestinal tract, kidney and urinary bladder, lung, male tissues, muscle tissues, pancreas, proximal digestive tract
Polypeptide *N*-acetylgalactosaminyltransferase 8 ^b)^	GALNT8	GalNAc [139]	Bone marrow and lymphoid tissues, brain, endocrine tissues, female tissues, gastrointestinal tract, kidney and urinary bladder, lung, male tissues, skin
Polypeptide *N*-acetylgalactosaminyltransferase 9	GALNT9	GalNAc [140]	^a)^
Polypeptide *N*-acetylgalactosaminyltransferase 10	GALNT10	GalNAc [141]	Adipose and soft tissue, bone marrow and lymphoid tissues, brain, endocrine tissues, female tissues, gastrointestinal tract, kidney and urinary bladder, lung, male tissues, muscle tissues, pancreas, proximal digestive tract, skin
Polypeptide *N*-acetylgalactosaminyltransferase 11	GALNT11	GalNAc [142]	Adipose and soft tissue, bone marrow and lymphoid tissues, brain, endocrine tissues, female tissues, gastrointestinal tract, kidney and urinary bladder, lung, male tissues, muscle tissues, pancreas, proximal digestive tract, skin
Polypeptide *N*-acetylgalactosaminyltransferase 12	GALNT12	GalNAc [143]	Appendix, bone marrow, brain, breast, cervix (uterine), endometrium, fallopian tube, prostate, soft tissue, thyroid gland, tonsil, skin
Polypeptide *N*-acetylgalactosaminyltransferase 13	GALNT13	GalNAc [144]	Adrenal gland, lung, salivary gland
Polypeptide *N*-acetylgalactosaminyltransferase 14	GALNT14	GalNAc [145]	^a)^
Polypeptide *N*-acetylgalactosaminyltransferase 15	GALNT15	GalNAc [146]	^a)^
Polypeptide *N*-acetylgalactosaminyltransferase 16	GALNT16	GalNAc [147]	Bone marrow and lymphoid tissues, brain, endocrine tissues, female tissues, gastrointestinal tract, kidney and urinary bladder, lung, male tissues, muscle tissues, pancreas, proximal digestive tract, skin
Polypeptide *N*-acetylgalactosaminyltransferase 17	GALNT17	GalNAc [148]	Brain
Polypeptide *N*-acetylgalactosaminyltransferase 18	GALNT18	GalNAc [149]	Adipose and soft tissue, bone marrow and lymphoid tissues, brain, endocrine tissues, female tissues, gastrointestinal tract, kidney and urinary bladder, lung, male tissues, muscle tissues, pancreas, proximal digestive tract, skin
Polypeptide *N*-acetylgalactosaminyltransferase like 5	GALNTL5	^c)^ [150]	Testis

^a)^ Only RNA expression data available in The Human Protein Atlas [16,17] and GeneCards [18] databases. ^b)^ FDA-approved drug target. ^c^^)^ Carbohydrate moieties recognized by this protein have not been discovered yet.

**Table 12:** Human X-type lectins, their carbohydrate ligands and protein expression in the organs.

Common Name (HUGO Name if Different)	Gene Symbol	Carbohydrate Preferential Affinity	Protein Expression in the Organs
Intelectin 1	ITLN1	Terminal acyclic 1,2-diol-containing structures, including β-d-galactofuranose, d-phosphoglycerol-modified glycans, d-glycero-d-talo-oct-2-ulosonic acid, 3-deoxy-d-manno-oct-2-ulosonic acid [174]	Appendix, colon, duodenum, rectum, small intestine
Intelectin 2	ITLN2	^a)^	Appendix, colon, duodenum, rectum, small intestine

^a)^ Carbohydrate moieties recognized by this protein have not been discovered yet.
